# Process Monitoring Evaluation and Implementation for the Wood Abrasive Machining Process

**DOI:** 10.3390/s101110401

**Published:** 2010-11-18

**Authors:** Daniel E. Saloni, Richard L. Lemaster, Steven D. Jackson

**Affiliations:** 1 Department of Forest Biomaterials, North Carolina State University, Campus Box 8005, Raleigh, NC 27695, USA; 2 Wood Machining and Tooling Research Program, Department of Forest Biomaterials, North Carolina State University, Campus Box 8005, Raleigh, NC 27695, USA; E-Mail: richard_lemaster@ncsu.edu; 3 Integrated Manufacturing System Engineering Institute, College of Engineering, Campus Box 7915 North Carolina State University, Raleigh, NC 27695, USA; E-Mail: steve_jackson@imsei.ncsu.edu

**Keywords:** abrasive machining, process monitoring, abrasive belt life, belt loading, acoustic emission, optical sensors

## Abstract

Wood processing industries have continuously developed and improved technologies and processes to transform wood to obtain better final product quality and thus increase profits. Abrasive machining is one of the most important of these processes and therefore merits special attention and study. The objective of this work was to evaluate and demonstrate a process monitoring system for use in the abrasive machining of wood and wood based products. The system developed increases the life of the belt by detecting (using process monitoring sensors) and removing (by cleaning) the abrasive loading during the machining process. This study focused on abrasive belt machining processes and included substantial background work, which provided a solid base for understanding the behavior of the abrasive, and the different ways that the abrasive machining process can be monitored. In addition, the background research showed that abrasive belts can effectively be cleaned by the appropriate cleaning technique. The process monitoring system developed included acoustic emission sensors which tended to be sensitive to belt wear, as well as platen vibration, but not loading, and optical sensors which were sensitive to abrasive loading.

## 1. Introduction

Abrasive machining is one of the most expensive processes in wood processing industries and therefore merits special attention and study. Abrasive machining processes are difficult to characterize and analyze because of the random nature and distribution of the grains on the abrasive belts. In the case of abrasive machining of a highly variable non homogeneous material such as wood, the complexity of the process increases and many variables have to be considered. The abrasive machining process represents an important investment in the machining process because machining belt price per unit is high and the belt life is short.

According to Stewart [1,2] the power required by a belt sander was positively correlated to the material removal rate, as well as with both depth of cut and feed rate of the wood being sanded. Other studies done by Saloni [3] showed that material removal rate was always higher when an aluminum oxide abrasive belt was used, in fact, sometimes nearly twice as high as other abrasive minerals. It was also found a correlation between the most critically controllable variables (interface pressure, wood species, rotational speed, grit size and abrasive mineral) in abrasive machining and the three main outputs: material removal rate, surface quality and power consumption. Multiple linear regressions described the general process and the effect of the variables on the outputs; however, large variability in surface quality and material removal rates were observed which demonstrated the complexity of the characterization [3].

Ratnasingam [4] discussed that it is possible to augment the production throughput by increasing the material removal rate; however, this will considerably increase the power consumption and reduce the life of the sanding belts. Moreover, they explained that this material removal rate increase will inevitably increase the temperature, which could result in an increase in loading, which has been proved to reduce the life of the belt by prematurely discharging the abrasive belts [4].

Two important factors that affect abrasive belt life are the loading of the belt and the belt temperature. These factors have been identified by many authors in the past and efforts have been directed toward developing feasible and effective solutions [5–7]. High temperatures have a considerable impact because increased temperature causes degradation of the abrasive belt and also cause problems in the workpiece (wood). The heat from the machining operation at the belt/wood interface can be so high that the heat melts resins in the wood. The liquefied resin then flows onto the belt, where the resin cools and become hard again, which result in loading. A loaded condition will cause the belt to be unable to cut additional material, as the grains cannot fully come into contact with the wood piece. The result is a situation where rubbing and friction generate excessive heat and the wood becomes burnished.

In addition, a reduction in the performance of the abrasive mineral accompanied clogging from material chips and adhesive particles within the grains for finer grit size [5]. High stock removal rates accelerated belt loading which can considerably shorten the belt life [8].

In addition, because the abrasive becomes loaded with wood fiber material, the material removal rates in abrasive machining can be reduced substantially [6]. This correlates to a reduction in machining efficiency as well as an increase in energy consumption. This cost is in addition to the high cost of abrasive belts (according to industry experts, many companies spend more than twice the cost of their sanding machine in purchasing abrasives annually).

There has been a great deal of research done to increase the life of abrasives. This ranges from developing new or different minerals, backing materials, and adhesives to methods of cleaning the abrasives. Many systems of abrasive cleaning are in existence in the woodworking industry today. One system manufactured by Nu-Life [14] uses a “Gum Eraser” block, which can be applied to a moving belt by manual or automated means (U.S. Patent 81/0081), or fed through the abrasive machine in sheet form. Other systems include pressure washing or chemical baths, including a cleaning system [15] that utilizes 8,274 kN/m^2^ and 60 °C water/cleaning solution. This system requires that the belt be removed from the machine and then placed in the bath for cleaning, which increases down time and labor costs. Other systems on the market have been developed to clean abrasives by blasting with either glass beads [16] or dry-ice crystals [17,18]. For purposes of this discussion, blasting is defined as the process by which particles are force impacting onto a surface for, in this situation, the purpose of cleaning.

High-pressure washing with or without detergent is the most common method, even though this technique is time consuming and requires the operator to stop the process to remove the machining belt from the machine.

Some research has been done in the past to determine the effect of cleaning on belt life. The usable life of the belt when cleaned can be increased by two to five times when belt cleaning is used for hardwood machining and considerably more when machining softwoods [6].

The implementation of cleaning techniques can increase or reduce the belt life and material removal rate depending on variables such as: technique used, blasting pressure, cleaning time, and intervals of cleaning [9]. In addition, a CO_2_ “snowflake” technique presented the best results for cleaning of abrasive belts [9].

Substantial research has been done to understand, and improve abrasive machining, covering the main pillars of the process such as machine configuration, abrasive belts and maintenance and cleaning methods [5–7]. However, integration of the systems of all of these pillars is required in order to consistently monitor the conditions of the process for further actions.

Process monitoring is a continuous real time activity used to determine the status of a process at any given time. The focus of monitoring is on the machine, the tools or tooling, the workpiece, or the process itself [10]. In addition, process monitoring is required to insure an optimum performance of manufacturing systems [10].

Lemaster and Dornfeld [11] demonstrated the feasibility of using acoustic emissions (AEs) to monitor the abrasive machining process. They showed that the AE technique was able to determine when the workpiece surface was smooth. The technique was also sensitive to belt wear and grit size as well as machining parameters such as feed speed and depth of cut. Additionally, they found that AE was not significantly sensitive to loading and cleaning.

AE can be used to monitor the abrasive machining process. Moreover, they established that AE was able to detect changes in material removal rates but not necessarily capable of monitoring abrasive loading or cleaning [12]. They concluded that this phenomenon was due to the removal of wood particle loading causing an increase in the penetration of the abrasive grains, which increase the generation of the AE. However, it also causes a decrease of the AE generation due to the friction between the wood specimen and the fact that loading is reduced [12].

The AE event count rates and the amplitude distribution of AE in sanding parallel to the grain were smaller and narrower than these values when sanding perpendicular to the grain. In addition, they determined that the AEs with low amplitude were greater for finer grit size and the AEs with high amplitude were greater for coarser grit size [13].

As stated above, sanding represents an important investment in the machining process because sanding belt price per unit is high and the abrasive belt life is short. It is not unusual to spend more than twice the cost of a machine for a year’s worth of abrasives. Any technique that could extend the life of the abrasives would be of great benefit to the woodworking industry and would result in energy savings, reduction in the number of abrasive belts required in addition to savings in labor and downtime due to belt replacement and setup.

In order to increase abrasive life, improve material removal rate, reduce down time and labor costs associated with the changing of the abrasive, and to reduce the overall number of abrasives that need to be purchased by a manufacturer, a multi-level research effort is needed. The objective of this study was to evaluate and demonstrate a process monitoring system for use in the abrasive machining of wood and wood based products.

## 2. Experimental

A series of tests were performed that measured loading and temperature during the sanding process. The following factors were selected to develop the experiment: wood composite (particleboard), abrasive (aluminum oxide), belt speed (3.62 m/s) and interface pressure (8,618 Pa). Abrasive mineral selection was based on the most common abrasive mineral utilized in the woodworking industry, which is aluminum oxide. The grit size selected was P100, based on wood industry practices and cited research [3]. The speed of the sanding belt was changed with a transmission attached to a 3-HP electric motor. The machine was designed to use standard 0.15 m × 1.22 m sanding belts of various grit sizes and minerals. A series of weights controlled the sanding pressure.

### 2.1. Optical Sensors

Belt loading was analyzed and monitored using various optical measurement techniques. These included three sensor types including: a CCD camera, an intensity detector [19] and an optical contrast detector [20]. The contrast detector determines the average gray scale intensity of the surface. This type of detector is inexpensive and eliminates the need for image analysis software (which is needed by the camera system). The disadvantage of the contrast detector is that it has to be placed very close (within 0.01 m) to the surface being evaluated. The intensity detector can have an offset distance up to 0.20 m. This would make the implementation of the intensity detector much easier in an industrial environment. Additionally, a ColorMax-1000 color sensor from EMX Industries, Inc. [21] was selected for analysis and testing.

**Figure 1.**
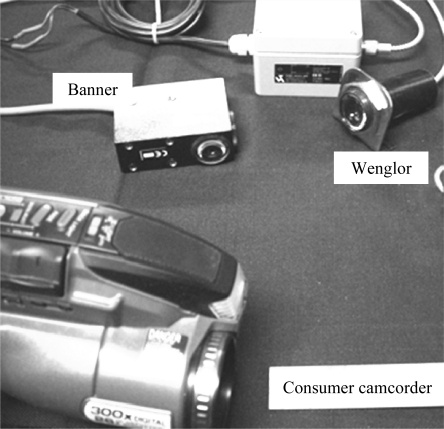
Sensors for determination of wood loading resulting from abrasive machining (clockwise from lower left: consumer camcorder, Banner® contrast detector and Wenglor® intensity detector).

Figure 1 shows a photograph of the three types of sensors, including two types of CCD cameras (a standard consumer camcorder and an industrial black and white machine vision camera), an intensity detector, and a contrast detector.

Different types of sensors were studied and analyzed in order to identify the best combination of sensors for monitoring loading and temperature, thus, optical sensors (color and gray scale) were used to monitor abrasive loading and IR thermometers were used to monitor temperature.

Optical sensors are inexpensive and easy to use and can be utilized to detect the loading on abrasive belts. Most of the optical sensors evaluated, however, exhibited a lack of accuracy and high variability in the measurements. Based on the preliminary results, the Banner^®^ sensor was eliminated due to its requirement of having to be close to the object to be measured. The CCD cameras were also analyzed but were rejected due to the requirement for post processing analysis of the signal.

The ColorMax 100 sensor was able to detect the formation of loading by exhibiting a considerable increase in the red color (the red color was the most significant and sensitive to change). The ColorMax 1000 sensor was slightly more sensitive in detecting the changes in the loading level when machining pine than particleboard. However, the ColorMax 1000 sensor signal did not appear to be sensitive to different loading levels during the abrasive machining process. Another shortcoming of the ColorMax 1000 sensor is that it requires the sensor to traverse the belt continuously in order to correctly monitor the status of the belt loading during abrasive machining. This is a result of the fact that localized loading occurs more often than uniform loading. It was concluded that the color sensor did not have enough sensitivity to the changes in loading to be of use in the evaluation of loading.

Results from the preliminary experimentation also showed that the Wenglor® intensity detector appeared to be the best option among the optical sensors studied. It offered the lowest experimental variation, and no required post processing of the output (such as image analysis) was required.

### 2.2. Acoustic Emission Sensors

Acoustic Emission (AE) and vibration sensors have been used extensively to monitor tool wear and tool life for cutting tools. Abrasive belt life was monitored by using acoustic emission sensors [5–7]. For this research, a D9241A that has a high sensitivity, low resonance frequency, low noise sensor (peak sensitivity 82 [–70] dB and operating frequency range 20–180 kHz) was utilized after preliminary research, with a wide range acoustic emission sensor (20 kHz − 1,000 kHz), identified the appropriate peak frequency to be used.

### 2.3. Temperature Sensors

The use of thermal-shock techniques to effectively clean abrasive belts as well as the appropriate combination of variables such as cleaning time, cleaning interval times and blasting pressure has been studied [7]. In addition, the life of the belt was considerably improved when the appropriate variables levels were used while the incorrect selection of levels could actually damage the abrasive belt [7]. Additionally, a comparison of different abrasive belt cleaning techniques such as dry ice pellets blasting, CO_2_ snowflakes blasting and walnut flour blasting has been discussed. They found that the best technique for removing loading on abrasive belts under the conditions used was CO_2_ snowflakes which was the abrasive cleaning technique used for this research to show the performance of the monitoring system designed and developed [9].

An experiment was designed to measure the interface contact temperature between the workpiece and the abrasive belt. To perform this experiment, a ThermoVision® A20V infrared camera by Flir™ [22] was utilized. This camera measures thermal energy emitted from an object (in this case, the energy emitted from the belt). The camera measures temperature ranges of −20 °C to +250 °C (−4 °F to +482 °F), and +120 °C to +900 °C (+248 °F to +1652 °F). The accuracy (% of reading) is ±2 °C or ±2%. The camera provides precise non-contact temperature measuring capabilities.

## 3. Results and Discussions

Results from this research are presented and discussed next in order to show the advantages and applications of process monitoring to continuously detect loading when abrasive machining is performed.

### 3.1. Optical Sensor

**Figure 2.**
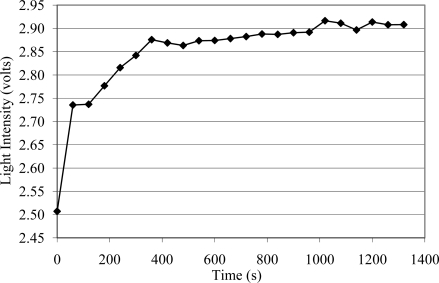
Wenglor® optical sensor signal as a function of machining time when abrasive machining is performed.

Figure 2 illustrates the light intensity from a Wenglor® sensor when machining particleboard using an aluminum oxide P100 belt at a 3.62 m/s belt speed. As illustrated in Figure 2, a continuous increase of the light intensity signal during abrasive machining occurs with the progression of belt loading. As can be seen, the loading formed rapidly and then continuously increased with time until a maximum level was reached. Moreover, preliminary research also showed that there is no significant difference in the signal when actually machining versus when the machine is idling (no machining).

**Figure 3.**
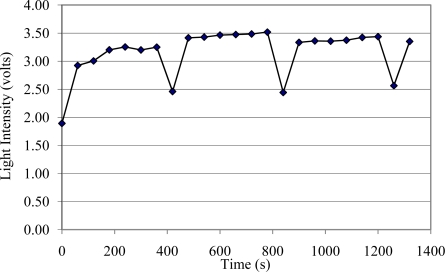
Wenglor® optical sensor signal after cleaning with CO_2_ flakes every 400 seconds during abrasive machining.

Figure 3 shows the intensity sensor signal (measured based on the voltage of the output signal) when cleaning with CO_2_ flakes for five seconds every 360 seconds after machining white pine with an aluminum oxide P150 belt. Figure 3 also shows that the light intensity signal continuously increased after the cleaning process was applied. In addition, it can be seen that the level of the light intensity signal was lower when the belt was new. Additionally, the “cleaned” light intensity level (level after cleaning was applied) tended to be similar regardless of the status of the belt or the level of the loading prior to cleaning. Thus, even though the loading level for second 660 was higher than second 1,020, the loading level after cleaning tended to be similar. The condition of the belt after each cleaning cycle never did reach the initial level (new belt). Finally, the “saw tooth” shape of the curve observed in Figure 3 clearly indicates the ability of the sensor not only to detect loading during the machining process after cleaning, but also the sensor’s capacity to monitor the cleaning process. It is important to note that the loading slope after cleaning tended to decrease, thus, the slope of the loading tendency from seconds 360 to 660 was larger than from seconds 720 to 1,020. It is speculated that this behavior is due to the abrasive grains becoming worn, which reduces the capacity of the grains to remove material causing a reduction in the amount of loading that can be accumulated. This would produce a larger difference in signal strength between a cleaned and non-cleaned belt. This effect would facilitate not only the detection of loading but would also indicate if the belt is getting worn due to mineral becoming dull. Thus, this would help the implementation of a system to control loading. Further investigation of the behavior of the loading and the effectiveness of cleaning techniques was conducted as is discussed below. In summary, the Wenglor® intensity sensor was shown to be effective in monitoring loading during the abrasive machining process. Thus, this sensor was used as a tool to continuously monitor loading and initiate associated process control actions (belt cleaning).

### 3.2. Temperature Sensor

**Figure 4.**
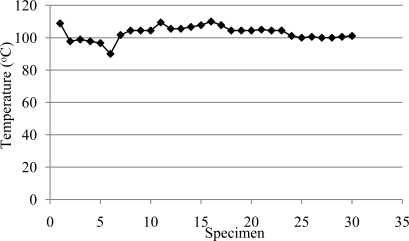
Interface temperature between workpiece and abrasive belt as measured by a thermographic A20V camera.

Temperature sensors have been used in a wide variety of industries to monitor different processes. The combination of heat and pressure from the abrasive machining operation at the belt/wood interface can be so high that the heat melts resins in the wood, which cools, and become hard again, which results in belt loading. A loaded condition will cause the belt to be unable to cut properly, as the grains cannot fully come into contact with the workpiece, causing rubbing and friction, which generates excessive heat, belt wear and workpiece surface problems. Thus, temperature can theoretically be used for the process monitoring, however, results indicated (Figure 4) that there was no significant change in temperature while performing the abrasive machining (increases in loading and tool wear). It was observed that after the first specimens machined, the temperature rapidly stabilized and slowly increased showing no clear indication of correlation between the number of specimens machined (more loading and belt wear) and belt temperature. This is a clear indication of the lack of sensitivity that the temperature sensors tested had for the detection of loading or abrasive belt life.

Thus, temperature sensors are not viewed as effective indicators of abrasive life and loading formation, however, they were found to be effective in helping establish high temperature limits in order to avoid burning the wood surface and / or belt damage.

### 3.3. Sensor Analysis and Selection to Monitor Abrasive Belt Life

Results showed the evaluation of AE sensors indicating that these sensors could be used effectively to monitor abrasive belt life (note that there was no indication that AE could effectively detect loading). It is important to note that the selection of the appropriate frequency band of the AE signal for monitoring the abrasive machining process was a critical consideration and was determined with a wide range acoustic emission sensor.

**Figure 5.**
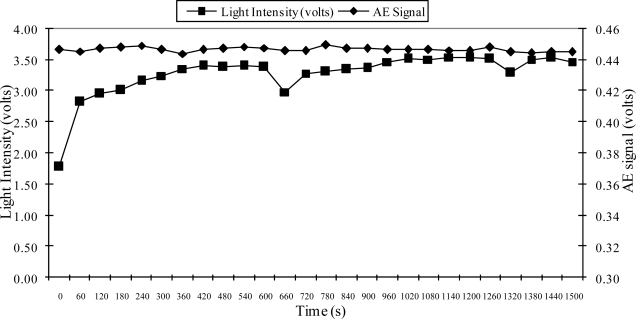
Wenglor® optical sensor and resonant acoustic emission sensor signals comparison when cleaning with CO_2_ flakes during abrasive machining.

Figure 5 showed that the acoustic emission signal was not detected by the cleaning process; since the signal did not show a significant change with increased loading. However, the loading was clearly observed by the optical sensor as indicated by a continuous increase of the signal from seconds 60 to 600 and from 600 to 1,200.

**Figure 6.**
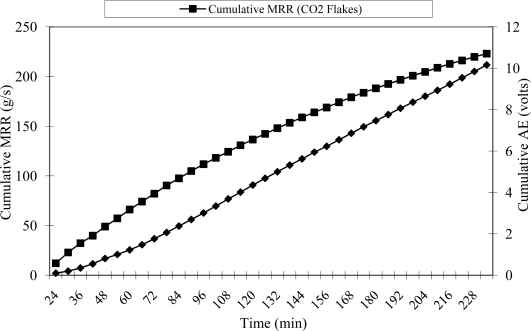
Cumulative material removal rate and cumulative contact resonant AE signal after cleaning with CO_2_ flakes during abrasive machining.

Figure 6 showed that the contact resonant AE sensor was able to measure belt wear with the appropriate location even for the belt speeds experienced during machining (43.33 ft/s–3.62 m/s).

Belt life was defined by using the output from the contact resonant AE sensor. Analysis of the acoustic emission output showed that the signal tended to increase until the signal stabilized at a certain level. Thus, a cumulative representation of the data was used for better understanding and analysis of the acoustic emission signal. The threshold acoustic emission level was defined primarily by defining the life of the belt using the material removal rate (MRR) which continuously decreases during the life of the belt until it tends to zero. For purposes of this research, the cumulative MRR was used and compared to the acoustic emission cumulative output in order to verify and validate the threshold for the belt life (Figure 6). Figure 6 shows that the slope of the cumulative material removal rate tended to decrease with time while the slope of the cumulative acoustic emission signal tended to increase with time. Thus, it was possible to define the threshold of the belt life based on the slope of two consecutive points when the cumulative acoustic emission data was greater than 3.5 for at least two consecutive measurements.

## 4. Conclusions

This research addressed the use of process monitoring and control techniques. However, no cleaning techniques were actually controlled based on the sensor output in this paper. Part II of this study evaluated the use of the proposed process control technique for improving productivity and reducing costs for abrasive machining applications in the wood products industry and will be reported in a subsequent manuscript.

The research included a literature review of abrasive machining and an experimental program which provided a solid background in the various aspects of abrasive machining, including; the abrasive machining process as related to wood products, abrasive belt wear mechanisms for woodworking applications, the design of abrasive belts for these applications, the characteristics of various sensors which could be useful in monitoring abrasive machining processes (including acoustic emission, optical, and thermal sensors), and the use of belt cleaning techniques for extending belt life by removing belt loading.

Based on the background research and experimental investigation, a process monitoring and control system that provided online detection of belt loading, belt wear, and belt/workpiece interface temperature was designed and developed into a working prototype system. This system used a combination of sensors to provide a reliable method of assessing belt loading and belt life.

The process monitoring and control system design was verified and refined in the laboratory for both the process monitoring and the process control (cleaning system) components. The laboratory prototype version of the process monitoring resulted in a substantial improvement in belt life and a reduction in the use of the blasting media (which would result in a major cost reduction for industrial users of abrasive belts).
